# Rehabilitation robotics in routine care: a minimum dataset and reporting framework for service delivery models

**DOI:** 10.3389/frhs.2026.1810720

**Published:** 2026-04-29

**Authors:** Rocco Salvatore Calabrò, Andrea Calderone

**Affiliations:** Department of Neurorehabilitation, IRCCS Centro Neurolesi Bonino-Pulejo, Messina, Italy

**Keywords:** health economics, health services research, health technology assessment, implementation science, rehabilitation robotics, reporting standard, service delivery model

## Abstract

Rehabilitation robotics has accumulated evidence for improving motor outcomes, yet adoption in routine care remains uneven across services and health systems. This evidence-to-practice gap reflects not only clinical considerations but also variation in how robotic rehabilitation is organized and delivered. Relevant service features include staffing and supervision patterns, scheduling rules, device placement, maintenance and support arrangements, governance, and documentation workflows. Current studies often describe the technology and clinical protocol while reporting service delivery features inconsistently, which limits transferability and weakens interpretation of implementation and economic findings. This perspective proposes a pragmatic reporting standard for service models in robotic rehabilitation. Its purpose is to make delivery configurations measurable and comparable across settings, while distinguishing the steady-state service delivery model from the time-limited implementation strategies used to establish, adapt, or sustain it. The standard includes a taxonomy of common service delivery archetypes, a Minimum Service Model Dataset for Rehabilitation Robotics (MSMD-RR) specifying must-report variables with operational definitions and units, and a reporting checklist, robotic rehabilitation service reporting (ROBOT-SERV), designed to complement established implementation science and health economic reporting guidance. MSMD-RR variables were selected for feasibility in routine care, cross-context interpretability, and plausible links to key drivers of real-world value, particularly utilization hours, throughput, therapist time, and downtime. A service model logic model is also provided to link inputs and processes to outputs and implementation, organizational, and economic outcomes. The proposed standard aims to support benchmarking, pragmatic evaluation, and health technology assessment. Graphical abstract

## 1 Introduction

Rehabilitation robotics has expanded rapidly over the last two decades, supported by trials and syntheses showing improvements in motor impairment and function for selected indications when delivered as part of structured rehabilitation programs (1–4). Nevertheless, routine implementation remains inconsistent across services and health systems, with marked variability in where, how, and by whom robotic rehabilitation is delivered. Diffusion of complex innovations in healthcare is rarely linear, and adoption is shaped by organizational capacity, professional roles, workflow fit, perceived value, and system incentives as much as by efficacy evidence (5, 6). Robotic rehabilitation makes these dynamics visible because capital investment, maintenance requirements, training needs, and governance obligations are substantial, while reimbursement and capacity benefits vary widely.

Implementation science has emphasized that the outcomes required to understand real-world uptake differ from clinical effectiveness outcomes and require explicit measurement and reporting (7). Economic conclusions are similarly context sensitive because transferability across jurisdictions depends on local cost structures, service configuration, and patterns of use (8). Budget impact and affordability decisions may diverge from cost-effectiveness in settings where upfront costs, utilization thresholds, and staffing constraints dominate (9, 10). Robotic rehabilitation is best understood as a complex intervention delivered within a complex adaptive system. Frameworks such as the Consolidated Framework for Implementation Research (CFIR) and the Reach, Effectiveness, Adoption, Implementation, and Maintenance (RE-AIM) framework highlight that characteristics of the intervention, inner and outer setting, implementation processes, reach, adoption, and maintenance can all influence real-world impact (11–14). Normalization Process Theory (NPT) further clarifies that sustained integration depends on sense-making, engagement, enactment, and appraisal by the people who deliver and receive care (15). These frameworks are widely used, yet the practical reporting of robotic rehabilitation service models often lacks the operational detail needed to map determinants to outcomes and to support transferability.

Intervention reporting standards, quality improvement reporting guidance, and economic evaluation reporting checklists offer strong foundations, but none are designed to capture the specific operational levers that characterize robotic rehabilitation in routine care (10, 16–19). Donabedian's structure-process-outcome framing provides a simple organizing principle, yet robotic rehabilitation frequently suffers from incomplete descriptions of structure and process components that drive outcomes beyond the patient level (20). The proposed standard is therefore scoped to the reporting problem that sits between device description and outcome measurement. Its purpose is not to prescribe a single optimal model but to standardize how models are described so that learning can accumulate across settings and decision-makers can interpret findings with greater confidence. This perspective uses the term “service delivery model” to denote the relatively stable configuration by which robotic rehabilitation is delivered in routine care. The model includes staffing and supervision patterns, scheduling rules, device placement such as hub-based or distributed deployment, maintenance and support arrangements, and governance and documentation workflows. Implementation strategies refer to the time-limited actions used to establish, adapt, or sustain a given service delivery model. Examples include training and competency programs, workflow redesign, audit and feedback, and stakeholder engagement. Separating the delivery configuration from the change process clarifies what should be measured as the service model and what should be reported as implementation work.

**Figure 1:**
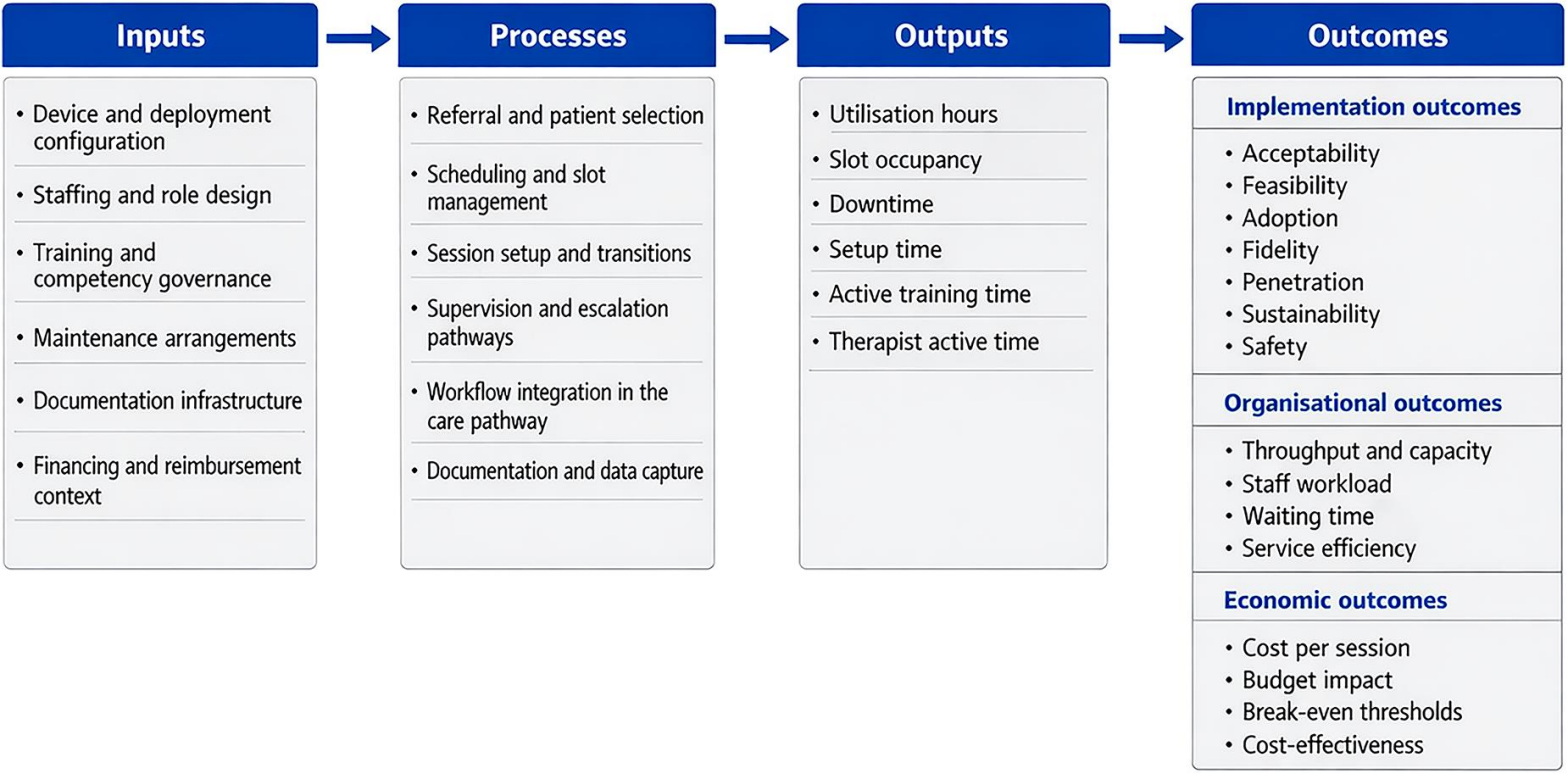
Service-model logic for robotic rehabilitation in routine care.

The standard has five components. The service archetype taxonomy is intended to classify organizational delivery configurations rather than devices themselves. Minimum Service Model Dataset for Rehabilitation Robotics (MSMD-RR) is proposed as a service-level minimum dataset for routine robotic rehabilitation delivery. The Robotic Rehabilitation Service Reporting (ROBOT-SERV), a pragmatic reporting checklist for robotic rehabilitation service delivery, is focused on service structure, workflow, utilization, reliability, staffing, and financing. These elements are novel in their combination and service-level scope. They are adapted conceptually, rather than derived *de novo*, from existing reporting and evaluation frameworks (21, 22). The Template for Intervention Description and Replication (TIDieR) remains focused on intervention description and replication. The Standards for Reporting Implementation Studies (StaRI) and the Standards for Quality Improvement Reporting Excellence (SQUIRE 2.0) address implementation and service improvement reporting. The Consolidated Standards of Reporting Trials (CONSORT) extension for pragmatic trials strengthens usual-care trial reporting. The Consolidated Health Economic Evaluation Reporting Standards (CHEERS 2022) strengthen economic reporting. The present proposal complements these frameworks by making operational determinants of routine robotic rehabilitation delivery explicitly measurable and comparable. A service model logic model links inputs, processes, outputs, and outcomes to support benchmarking, pragmatic evaluation, and health technology assessment (Figure 1). Most robotic rehabilitation studies describe device class, training dosage, and patient outcomes, but report service organization inconsistently (23–26). The present framework shifts the reporting focus to delivery configuration, staffing model, supervision ratio, setup burden, utilization, downtime, documentation, and financing. It is therefore positioned not as a replacement for existing standards, but as a service-model layer that sits between device description and outcome reporting. This positioning is intended to make the framework usable not only in dedicated service-level studies but also in the much larger body of studies primarily focused on the feasibility, acceptability, or effectiveness of a specific robotic intervention. In these contexts, the proposed standard functions as a pragmatic contextual overlay rather than as a competing reporting scheme. A study may remain centered on patient-level clinical outcomes, device performance, or intervention feasibility, while additionally reporting the service configuration in which the intervention was delivered. This includes, at minimum, the staffing model, supervision ratio, deployment configuration, setup burden, active training time, therapist active time, utilization, downtime, documentation workflow, and reimbursement context. These variables help readers distinguish whether observed results are likely to reflect the intrinsic intervention, the surrounding delivery model, or an interaction between the two. They therefore improve interpretability, transferability, and the credibility of implementation and economic inferences without displacing the primary clinical focus of the study.

## 2 Service delivery model taxonomy

**Figure 2:**
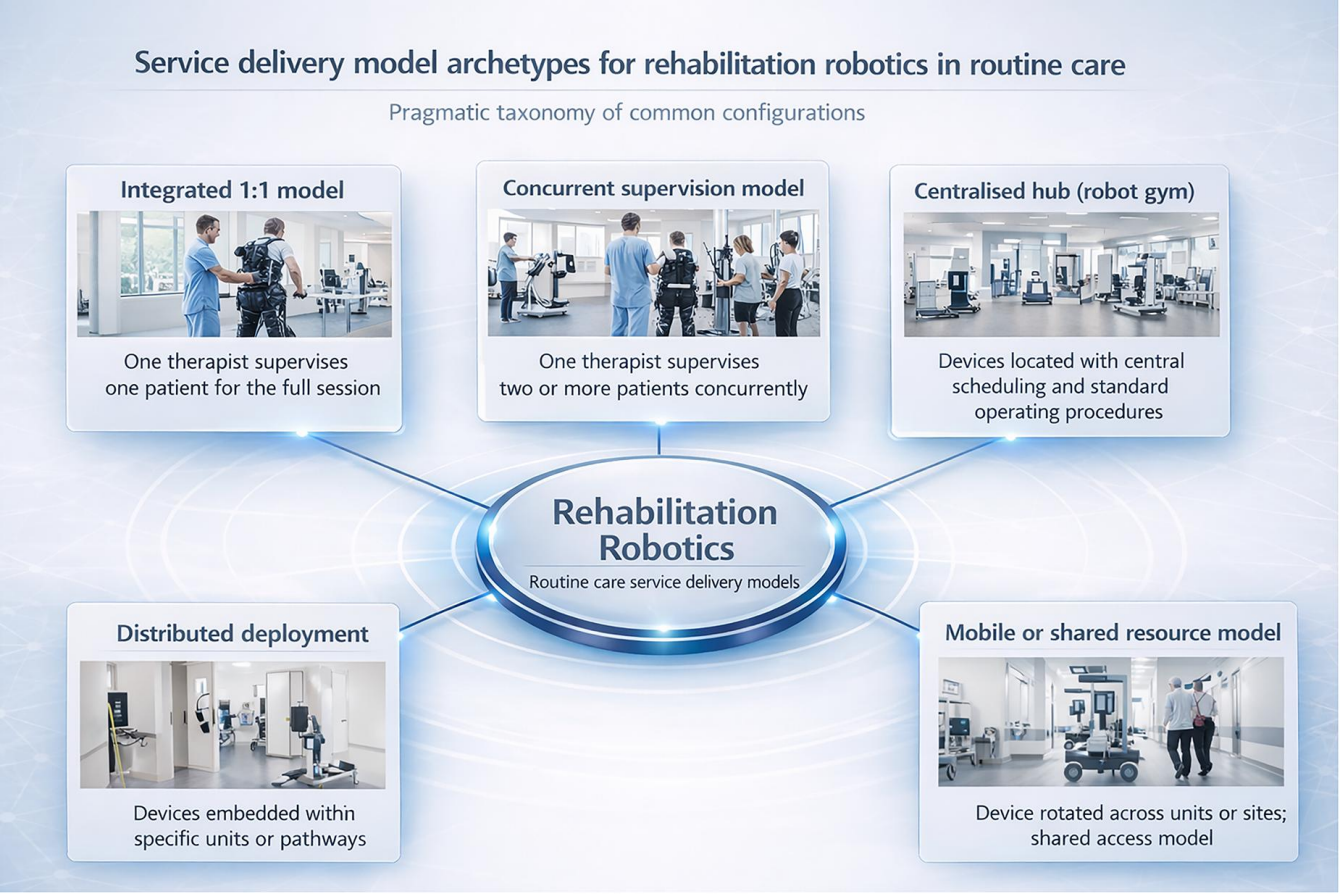
Conceptual taxonomy of rehabilitation robotics service delivery models in routine care. The figure summarizes five pragmatic service archetypes: integrated 1:1 model, concurrent supervision model, centralized hub (robot gym), distributed deployment, and mobile or shared resource model, highlighting differences in supervision structure, device allocation, and workflow organization.

Service delivery models for robotic rehabilitation vary along a small number of defining dimensions, even when clinical protocols appear similar. Supervision ratio and staffing mix influence therapist time per session and determine whether robotic rehabilitation expands capacity or simply substitutes for conventional therapy. The physical location of devices, whether concentrated in a dedicated space or distributed across clinical units, shapes scheduling, transport time, and integration with multidisciplinary pathways. Governance arrangements and the presence of dedicated roles, such as a clinical champion or a technician, influence reliability, safety, and continuity. These dimensions can be combined to define a pragmatic taxonomy of archetypes that are recognizable across services (Table 1; Figure 2). These archetypes are intended to span upper-limb robots, robotic gait training systems, and wearable exoskeletons. The feasibility of concurrent supervision and the magnitude of setup time will vary by device class and patient acuity, so classification should be paired with reporting of the defining operational variables in MSMD-RR.

The first archetype is an integrated one-to-one model, in which robotic sessions are delivered similarly to conventional therapy, typically with one therapist supervising one patient for the full session. This model often emerges during early adoption, supports close tailoring and safety monitoring, and may be preferred for patients requiring high assistance. Its limitations relate to opportunity cost when therapist time remains the binding constraint and to reduced incentives for scaling up if the robot does not increase throughput.
A second archetype is a concurrent supervision model, in which one therapist supervises two or more patients in parallel, each using a robotic device or a combination of devices and independent exercises. This model treats the robot as a capacity multiplier, and its feasibility depends on patient selection, device usability, safety features, and room layout. Supervision ratio is the defining parameter, but the model also requires explicit workflow design to manage setup, transitions, and documentation without compromising fidelity or safety.
A third archetype is a centralized hub, often described as a robot gym model. In this configuration, multiple devices are collocated with dedicated scheduling and standard operating procedures, often with a support role responsible for setup, troubleshooting, and maintenance coordination. Centralization can improve utilization and reduce downtime through standardization, while also enabling training and competency maintenance. Trade offs include patient transport burden and potential disconnection from ward-based workflows unless pathways are designed to integrate the hub.
A fourth archetype is distributed deployment, where devices are embedded within specific clinical areas, such as an inpatient unit, an outpatient gym, or a community service. This can improve integration with local teams and reduce transport delays while limiting the need for a dedicated hub. Resource duplication and uneven utilization are common risks, and the model often requires stronger governance to ensure consistent training, maintenance, and safety reporting across multiple locations.
A fifth archetype is a mobile or shared resource model. In this configuration, a device is moved between units or sites or used in rotational blocks across services. This approach can reduce capital barriers and support network-level access, but it increases logistical complexity and may increase downtime, setup time, and variability in staffing competence. Documentation systems, responsibility for consumables, and maintenance escalation routes must be defined to prevent fragmentation.

**Table 1:** Service delivery model taxonomy and defining features.

Archetype name	Core workflow	Supervision model	Key resource constraints	Typical context
Integrated 1:1 model	Robotic session delivered as a standard therapy appointment within usual scheduling	One therapist supervises one patient for the full session	Therapist time remains the primary bottleneck; limited capacity gain	Early adoption, high acuity patients, settings prioritising tailoring and close monitoring
Concurrent supervision model	Multiple patients complete robotic training in parallel, often combined with independent exercises	One therapist supervises two or more patients concurrently	Room layout, patient selection, device usability, safety procedures	Outpatient gyms, high volume services, programmes aiming to expand capacity
Centralised hub (robot gym)	Devices colocated in a dedicated area with standard operating procedures and central scheduling	Variable, often supported by a dedicated technician or assistant	Space allocation, patient transport, governance and scheduling coordination	Centres with multiple devices, services aiming to maximise utilisation and standardisation
Distributed deployment	Devices embedded within specific units or pathways, integrated into local team routines	Typically local team supervision with pathway specific processes	Risk of uneven utilisation; duplicated training and governance effort	Inpatient units, disease specific programmes, services prioritising pathway integration
Mobile or shared resource model	Device rotated across units or sites based on scheduled blocks or referrals	Variable, depends on local staff availability and competence	Logistics, setup variability, fragmented ownership and maintenance pathways	Networked services, limited capital, shared procurement or leasing arrangements

Taxonomies are useful only when they reduce ambiguity without oversimplifying reality. The proposed archetypes are intended as a primary classification that can be refined by reporting the key defining variables in the MSMD-RR. Services may transition between archetypes over time, and hybrid models will occur. A shared language remains valuable because it enables comparative learning, supports mapping of determinants to outcomes, and clarifies which aspects of a model are intended to drive value.

## 3 Minimum service model dataset (msmd-Rr)

The central contribution of this Perspective is the MSMD-RR, a minimum dataset of must-report variables that describe service delivery models with sufficient operational precision to support comparability (Table 2). The dataset is intentionally limited. It prioritizes variables that are both feasible to capture in routine services and plausibly linked to implementation, organizational, and economic outcomes. Measurement burden must remain proportionate, or reporting standards will fail to be adopted. In the present proposal, the irreducible minimum is the set of variables that should be reported in any routine-care evaluation of robotic rehabilitation services. Additional variables should be treated as an extended set and reported when feasible to enrich contextual, implementation, and economic interpretations.

**Table 2:** MSMD-RR: minimum variables, operational definitions, and measurement units.

Domain	Variable	Operational definition	Recommended unit and data source
Context and pathway	Setting and phase of care	Care setting and phase in which sessions occur, including inpatient, outpatient, community, and acute, subacute, or chronic pathways	Categorical; service records and pathway documentation
Context and pathway	Clinical population and indication	Primary diagnosis group and functional indication for which the robot is used in routine care	Categorical; referral criteria and clinical records
Device and technical	Robot category	Device category relevant to operation, such as upper limb robot, gait robot, exoskeleton, or end effector	Categorical; device documentation
Device and technical	Deployment configuration	Physical placement and access model, including hub, distributed, or mobile deployment	Categorical; service configuration documents
Workforce and roles	Staffing mix	Roles involved in delivery, such as physiotherapist, occupational therapist, assistant, technician, and their presence during sessions	Categorical plus counts; rosters and session logs
Workforce and roles	Supervision ratio	Number of patients supervised per therapist during the active training period	Ratio; session logs and observation
Workforce and roles	Dedicated support role presence	Whether a named role exists for champion, super user, or technician support, and responsibilities	Categorical; role descriptions
Training and competency	Training dose	Initial training time per staff member for device operation and workflow	Hours per person; training records
Training and competency	Competency maintenance approach	Method used to maintain competence, such as refresher sessions, checklists, or sign off	Categorical; training governance documents
Workflow and scheduling	Scheduled session duration	Planned appointment length for robotic rehabilitation sessions	Minutes; scheduling system
Workflow and scheduling	Setup time	Time from patient arrival to initiation of active robotic training	Minutes; time motion sampling or structured documentation
Workflow and scheduling	Active training time	Net time during which the patient is actively engaged with the robotic training task, excluding setup and interruptions	Minutes; device logs where available or structured observation
Workflow and scheduling	Therapist active time	Therapist time spent on direct setup, hands-on assistance, and monitoring during the session	Minutes; time motion sampling or structured documentation
Utilisation and capacity	Utilisation hours	Total hours of active training delivered per device per year in routine care	Hours per year; device logs or session records
Utilisation and capacity	Slot occupancy and cancellations	Proportion of scheduled slots completed, and reasons for cancellations or non attendance	Percent plus categories; scheduling system
Reliability and maintenance	Downtime	Time the device is unavailable for use due to faults, maintenance, or other operational reasons	Hours per month or year; maintenance logs
Reliability and maintenance	Maintenance model	Type of maintenance arrangement, such as manufacturer contract, in house service, or pay per use agreement	Categorical; procurement and service contracts
Safety and governance	Adverse device events reporting	Definition and routine capture approach for adverse events and near misses related to device use	Categorical plus counts; incident reporting system
Data and documentation	Documentation integration	How session data and clinical notes are captured, including electronic health record (EHR) integration, stand alone documentation, or manual transcription	Categorical; clinical documentation workflow
Financing and reimbursement	Payment and reimbursement context	Reimbursement mechanism and whether robotic rehabilitation is reimbursed distinctly, bundled, or not reimbursed	Categorical; billing policies and payer rules
Financing and reimbursement	Cost accounting scope	Components included in costing, including capital, depreciation, maintenance, consumables, training, staff time, and overhead	Categorical; finance and costing systems

Context and pathway variables anchor interpretation. Variables were selected using three pragmatic criteria. Each variable must be observable in routine care with low additional burden, using sources such as scheduling systems, device logs, maintenance records, or standard clinical documentation. Each variable must also be plausibly linked to proximal drivers of real-world value, particularly utilization hours, throughput, therapist time, and downtime, which strongly influence cost per session and budget impact. Variables were prioritized when they were interpretable across device classes and service contexts, enabling comparison and synthesis. Setting, phase of care, clinical population, and pathway position determine patient acuity, staffing norms, and expected intensity. Device and technical variables describe robot type and key operational features that influence setup complexity, reliability, and suitability for concurrent supervision. Workforce variables include staffing mix, supervision ratio, and role definitions, because these determine therapist time per unit of delivered therapy and influence both fidelity and scalability. Supplementary Figure 3 provides an illustrative example of a multidisciplinary robotic rehabilitation environment, highlighting the staffing mix, support roles, and technology presence that shape routine-care service delivery. Supplementary Figure 4 provides a concise applied vignette contrasting an inpatient gait-robot hub with a distributed upper-limb robotic system and illustrates how core MSMD-RR variables and ROBOT-SERV items can be instantiated across two realistic routine-care service configurations.

Workflow variables require particular attention. Scheduled session duration is often reported, yet the dose that matters for motor learning depends on active training time, repetition, and task practice (27, 28). Set-up time and therapist active time, therefore, need explicit reporting, ideally using device logs, time motion sampling, or structured documentation. Utilization hours per year and slot occupancy are critical because cost per session is dominated by fixed costs when utilization is low. Downtime and cancellation rates provide information on reliability and operational stability and help distinguish whether limited utilization reflects demand constraints, staffing constraints, or technical failure.

Governance and safety reporting must be routine. Safety reporting should distinguish device-related events, including interface injuries and hardware malfunctions, from therapy-related events such as falls, overexertion, or symptom exacerbation. The reporting channel should be specified, for example, incident reporting systems, the electronic health record, or both. Adverse device events, near misses, and discontinuations should be defined and captured consistently, and protocols for eligibility, supervision escalation, and emergency stops should be referenced. Documentation variables are also essential because integration with electronic health records can influence therapist workload and data quality. Financing variables capture the reimbursement context, procurement approach, and maintenance contract type, because these shape incentives and determine whether the service model can be sustained. Transferability of economic evidence depends on explicit reporting of these features, not only on unit costs (8, 10).

The MSMD-RR is not a comprehensive dataset for all research questions. Its irreducible minimum is the set of variables required for baseline service description and comparison across settings: setting and phase of care, clinical population and indication, robot category, deployment configuration, staffing mix, supervision ratio, scheduled session duration, setup time, active training time, therapist active time, utilization hours, slot occupancy and cancellations, downtime, maintenance model, adverse device event reporting, documentation integration, payment and reimbursement context, and cost accounting scope. Candidate extended variables, to be reported when feasible, include dedicated support role presence, training dose, competency maintenance approach, referral-to-treatment interval, reasons for non-start, premature discontinuation, patient transport burden, repeated setup attempts, consumable use, calibration burden, and software-update burden. This separation supports uptake of the standard while allowing richer contextual, implementation, and economic reporting when data infrastructure permits.

## 4 Recommended outcomes set for service model evaluation

A standardized description of a service model is useful only if the outcomes used to evaluate it are aligned with its mechanisms. Implementation outcomes provide a necessary bridge between service design and sustained delivery. Proctor and colleagues distinguished key outcomes such as acceptability, adoption, appropriateness, feasibility, fidelity, coverage, sustainability, and implementation cost (7). These outcomes are particularly relevant for robotic rehabilitation because high perceived burden, weak workflow fit, or limited organizational readiness can prevent normalization even when clinical benefits are evident (15, 29).

Organizational outcomes should capture whether robotic rehabilitation changes service capacity, staff workload, and patient flow. Throughput and capacity metrics, such as sessions delivered per week, waiting time for therapy, and therapy intensity achieved, reflect whether the service model operates as intended. Donabedian's framing remains useful here because it emphasizes that outcomes depend on both structure and process and that structural investments can fail without process integration (20). Organizational outcomes also include unintended consequences, such as increased documentation burden, congestion in shared spaces, or inequitable access if selection criteria favor certain patient groups.

Economic outcomes should be selected based on the decision context. Cost per session and cost per unit of active training time provide operationally meaningful metrics and should reflect both fixed and variable costs. Budget impact analysis is essential for payers and providers because affordability often determines adoption (9). Cost-effectiveness remains relevant for longer-term value assessment, yet its conclusions depend on assumptions about utilization, staffing, and pathway effects that are directly influenced by the service model (8). The reporting standard therefore links the MSMD-RR variables to economic analyses by making key drivers observable rather than assumed.

Equity and reach deserve explicit attention. Implementation frameworks and trial reporting extensions have argued for routine consideration of differential access and impact (30). Robotic rehabilitation may widen inequities if access is concentrated in specialized centers, if eligibility criteria inadvertently exclude underserved populations, or if staffing and scheduling practices privilege certain pathways. Service model reporting that includes reach, pathway location, and patient flow can support equity-focused interpretation.

## 5 The robot-serv reporting checklist

ROBOT-SERV is proposed as a pragmatic checklist to support consistent reporting of robotic rehabilitation service models in routine care evaluations. The checklist is not intended to replace established standards. Instead, it complements and operationalizes them for the specific challenges of robotic rehabilitation. ROBOT-SERV distinguishes core items that should be reported in all evaluations from extended items that are recommended when feasible. Supplementary Figure 5 provides a rapid visual summary of this two-tier structure by separating essential items for any study or service evaluation from additional items recommended when local reporting capacity and data infrastructure permit. TIDieR improves the description of interventions and replication (16). StaRI supports transparent reporting of implementation studies (17). SQUIRE 2.0 guides reporting of quality improvement (18). CONSORT extensions for pragmatic trials improve reporting when effectiveness is evaluated under usual care conditions (19). CHEERS 2022 strengthens reporting of economic evaluations (10). ROBOT-SERV bridges these approaches by focusing on operational variables that are often treated as background. Yet these variables are core determinants of adoption, efficiency, and economic performance. In intervention-focused studies, these items can be reported as contextual descriptors of the delivery setting, allowing feasibility and effectiveness findings to be interpreted against the organizational conditions in which the robotic intervention was actually implemented.

The checklist is structured around the service model elements captured in the MSMD-RR and maps each reporting item to outcomes that it is expected to influence (Table 3). This mapping is important because it discourages reporting for its own sake and clarifies why each item matters. It also supports peer review and critical appraisal. A reader can assess whether a study's conclusions about feasibility or cost-effectiveness are credible given the reported utilization, downtime, staffing, and workflow integration. ROBOT-SERV distinguishes the steady-state service model from the implementation strategies used to establish, adapt, or sustain it in practice.

**Table 3:** ROBOT-SERV reporting items mapped to outcomes (core vs extended).

Reporting item	Rationale	Linked outcomes	Common data source	Tier
Service setting and pathway position	Enables interpretation of acuity, staffing norms, and expected intensity	Adoption, feasibility, penetration, throughput	Service pathway documents and clinical records	Core
Patient selection criteria and exclusions	Clarifies reach and potential inequities in access	Reach, penetration, equity, acceptability	Referral criteria and eligibility protocols	Core
Robot type and deployment configuration	Determines operational demands and integration opportunities	Feasibility, fidelity, downtime, utilisation	Device inventory and service configuration	Core
Staffing model and supervision ratio	Core determinant of capacity effects and cost per session	Throughput, staff workload, cost per session	Rosters, session logs, time studies	Core
Workflow description including setup and transitions	Prevents hidden variability in delivered dose and efficiency	Fidelity, efficiency, therapist time	Observation, process maps, structured documentation	Core
Training programme and competency maintenance	Influences safety, fidelity, and sustainability	Fidelity, safety, sustainability	Training records and governance	Core
Scheduling model and slot management	Drives utilisation, occupancy, and patient flow	Utilisation, penetration, waiting time	Scheduling system and referral lists	Core
Maintenance arrangement and escalation pathway	Determines downtime and resilience	Downtime, feasibility, sustainability	Maintenance logs and contracts	Core
Safety governance and incident reporting approach	Supports routine risk management and transparency	Safety, acceptability, sustainability	Incident reporting systems	Core
Documentation and data capture method	Affects staff burden and data quality for evaluation	Staff workload, fidelity, implementation cost	Electronic health records and local documentation tools	Core
Financing and reimbursement description	Essential for transferability and affordability decisions	Budget impact, sustainability, adoption	Billing policies and finance systems	Core
Implementation strategies used	Supports interpretation of change processes and replication	Adoption, penetration, sustainability	Implementation plans and strategy logs	Extended
Outcomes selected and time horizon	Aligns evaluation with decision context and mechanisms	Implementation, organisational, economic outcomes	Study protocol or service evaluation plan	Core
Contextual changes during implementation	Clarifies threats to validity and interpretation	Adoption, sustainability, organisational outcomes	Service reports and governance minutes	Extended

Implementation strategies should be specified and reported using established recommendations, because service redesign is itself an intervention (31). The Expert Recommendations for Implementing Change (ERIC) taxonomy provides a common language for strategies such as training, audit and feedback, workflow redesign, and stakeholder engagement (32). Behavioral and organizational mechanisms can be articulated through complementary frameworks, including the Behavior Change Wheel and the Theoretical Domains Framework, when professional behavior change and competency development are central to the implementation plan (33, 34). ROBOT-SERV does not mandate a single theoretical lens, yet it encourages explicit specification to support accumulation of knowledge.

## 6 Service model logic and intended uses

Figure 1 presents a service logic model that connects inputs, processes, outputs, and outcomes for robotic rehabilitation. Inputs include the device, maintenance arrangements, staffing, training, and governance structures. Processes capture workflow design, scheduling, supervision, documentation, and safety procedures. Outputs represent immediate operational performance, including utilization hours, slot occupancy, downtime, setup time, and therapist time. Outcomes include implementation outcomes, organizational effects, and economic results.

The logic model is intended to support three uses. Benchmarking is the first. Services can track core outputs and compare performance over time while interpreting changes in relation to staffing or workflow modifications. Pragmatic evaluation is the second. Research designs that aim to inform decision-making under real-world conditions depend on measurement of routine processes and outputs, not only clinical endpoints (35). Health technology assessment is the third. Decision makers require transparent reporting of the service model to interpret whether results are transferable and to understand the drivers of cost and benefit (8–10). The logic model also provides a structure for data extraction and evidence mapping in a subsequent scoping exercise that will characterize implementation determinants and strategies in rehabilitation robotics.

## 7 Implications for research, practice, and health technology assessment

A minimum dataset and reporting checklist can create shared infrastructure for learning. Routine capture of MSMD-RR variables would support registries and service monitoring and could enable multi-site comparisons even when randomization is not feasible. MSMD-RR is intended as a service-model layer that future registries could adopt, rather than as a substitute for clinical registries centered primarily on patient outcomes. Importantly, the framework is also designed to be usable within studies whose primary aim is to evaluate the feasibility, acceptability, or effectiveness of a specific robotic intervention. In such studies, the core MSMD-RR and ROBOT-SERV elements can be applied as a light-touch contextual layer to document the operational conditions under which the intervention was delivered. This makes it possible to interpret whether differences in feasibility, adherence, delivered dose, throughput, or cost are likely to reflect the intervention itself or the way the service was organized around it. Such reporting may also improve between-study comparability by reducing the frequent underdescription of staffing, supervision, utilization, workflow, and technical downtime. The proposed standard is therefore consistent with complex-intervention guidance and hybrid effectiveness-implementation designs because it makes service model components explicit and measurable (36–39).

Clinical services may benefit from a clearer articulation of roles and workflows. Organizational readiness for change and the presence of champions and support structures can determine whether new technologies become normalized (24, 40). Perceived usefulness and ease of use influence acceptance. Established models of technology acceptance therefore offer constructs that can inform training and workflow design (41–43). ROBOT-SERV provides a structure for documenting these determinants in a way that is relevant to service leaders and payers.

Health technology assessment in robotic rehabilitation requires attention to transferability. Economic evaluation methods recognize that costs and effects must be interpreted in context and that jurisdictional differences can invalidate naive extrapolation (8). Budget impact analysis provides an additional lens that is often decisive for adoption (9). By standardizing reporting of utilization, downtime, staffing, and financing, the proposed framework reduces reliance on untested assumptions and supports more credible assessments.

## 8 Limitations and boundary conditions of the proposed standard

The proposed standard has limitations. Empirical validation and consensus development have not yet been conducted, and the taxonomy and dataset may require refinement through stakeholder engagement and iterative testing. Some services may lack routine data infrastructure, and measurement burden could be a barrier if variables are interpreted as research only. A pragmatic implementation approach is therefore essential, beginning with a minimal subset and expanding as capability grows.

Robot heterogeneity also presents a challenge. Exoskeletons, end effector devices, and upper limb robots differ in safety profiles, setup demands, and suitability for concurrent supervision (44). The MSMD-RR therefore focuses on operational variables that remain meaningful across devices while allowing device-specific extensions. Reimbursement and regulatory environments vary across countries, and definitions of “session,” “supervision,” and “documentation” may differ. The standard should be applied with clear local definitions, and interpretation should explicitly consider the outer setting, consistent with implementation theory (11, 27).

## 9 Conclusion

Robotic rehabilitation is increasingly evidence-based, yet translation into routine care depends on service delivery design as much as on device capability. Current reporting often leaves the service model implicit, limiting transferability and slowing accumulation of actionable knowledge. This Perspective proposes a pragmatic standard comprising a service model taxonomy, a Minimum Service Model Dataset for Rehabilitation Robotics, and a reporting checklist, ROBOT-SERV, supported by a logic model linking operational outputs to implementation, organizational, and economic outcomes. Adoption of this standard could improve comparability across centers, strengthen pragmatic evaluation, and provide a structured foundation for a future mapping review of determinants and implementation strategies in rehabilitation robotics.

## Data Availability

The original contributions presented in the study are included in the article/Supplementary Material, further inquiries can be directed to the corresponding author/s.
