# Supplementary figures and images for: Rehabilitation robotics in routine care: a minimum dataset and reporting framework for service delivery models
Source: Front Health Serv. 2026 Apr 29;6:1810720. doi: 10.3389/frhs.2026.1810720 (PMC13168165; doi:10.3389/frhs.2026.1810720)

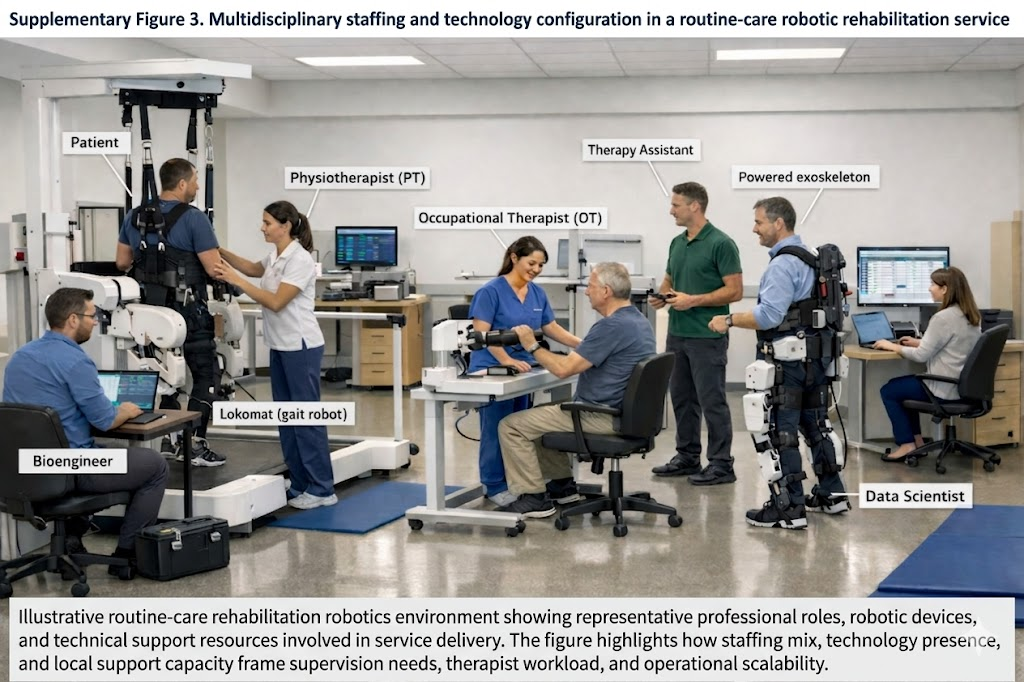

Supplement: Supplementary file 1 [file Image1.tiff]

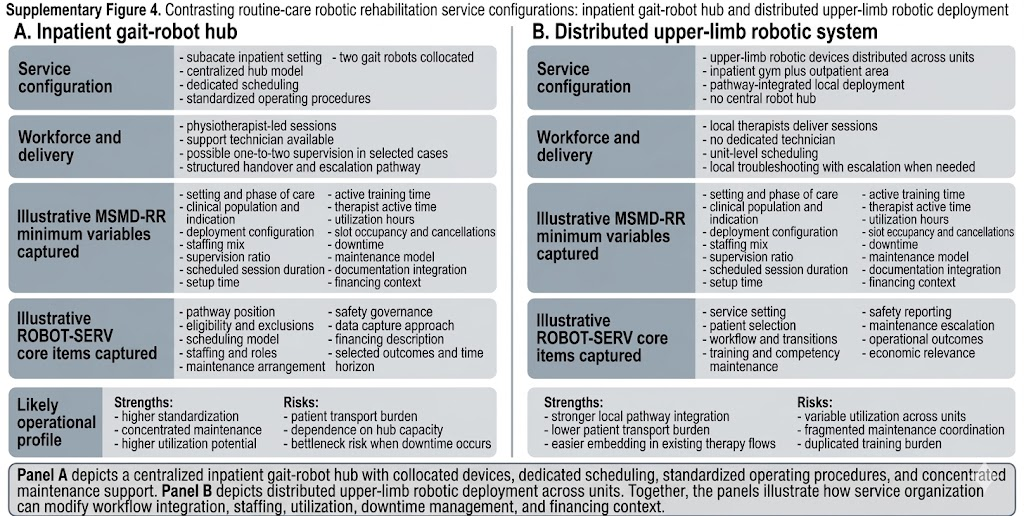

Supplement: Supplementary file 2 [file Image2.tiff]

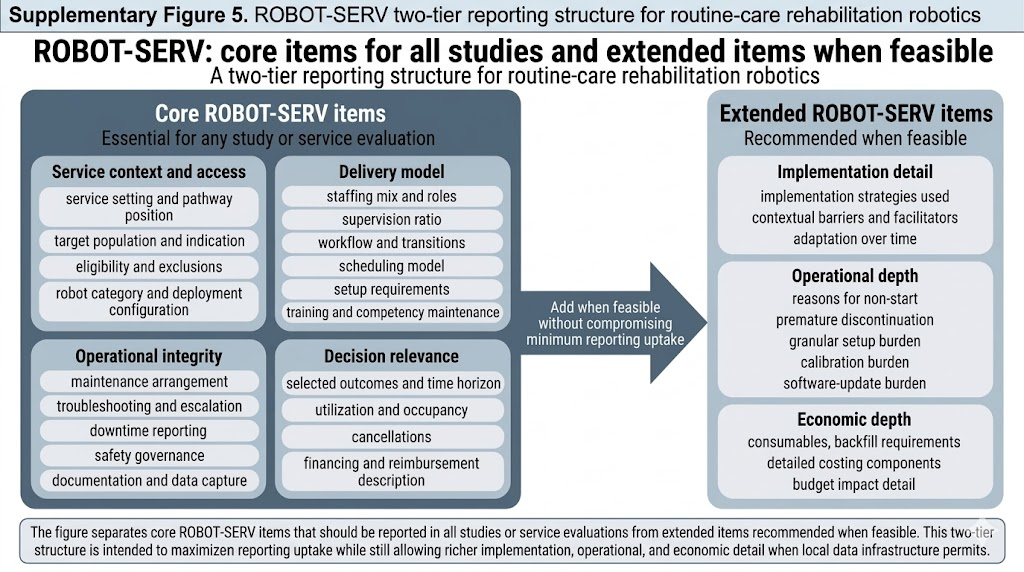

Supplement: Supplementary file 3 [file Image3.tiff]

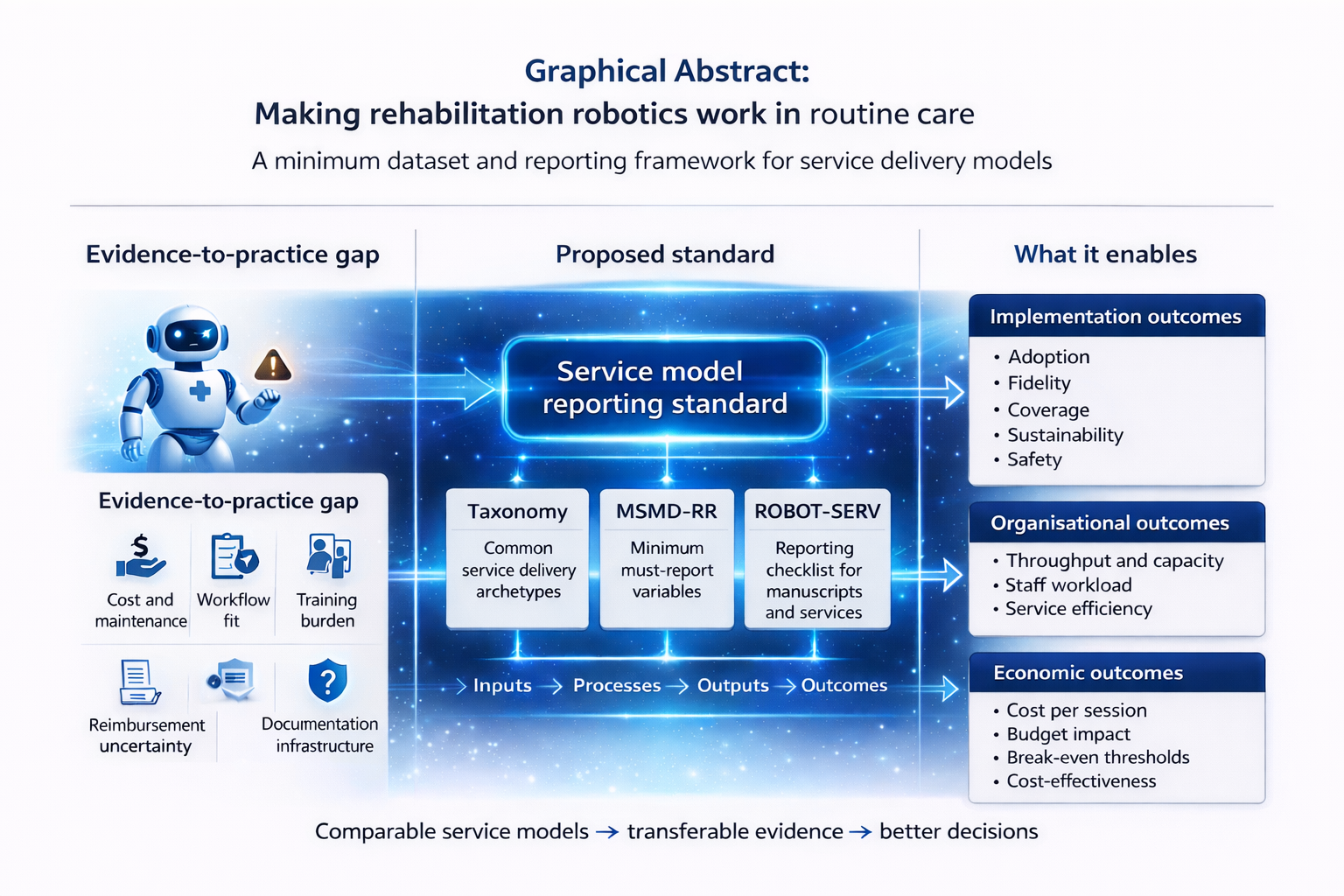

Supplement: Supplementary file 4 [file Image4.tiff]
